# Esophageal perforation in case of thyroid lymphoma

**DOI:** 10.1002/ccr3.2858

**Published:** 2020-04-14

**Authors:** Panagiotis Anagnostis, Konstantina Vaitsi, Keraso Tzelepi, Efstratios Kalaitzis

**Affiliations:** ^1^ Department of Endocrinology Police Medical Center of Thessaloniki Thessaloniki Greece; ^2^ Panorama Surgery Center ‘Aghios Loukas’ Hospital Thessaloniki Greece

**Keywords:** compressive symptomatology, esophageal perforation, neck enlargement, thyroid lymphoma, tracheal compression

## Abstract

Esophageal perforation is a rare and serious complication of thyroid lymphoma and should be taken under consideration in cases with rapid deterioration in their course. Clinical suspicion and prompt diagnosis are key factors for reducing mortality risks in these cases.

## Graphical abstract

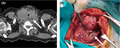

## 1 INTRODUCTION

**FIGURE 1:**
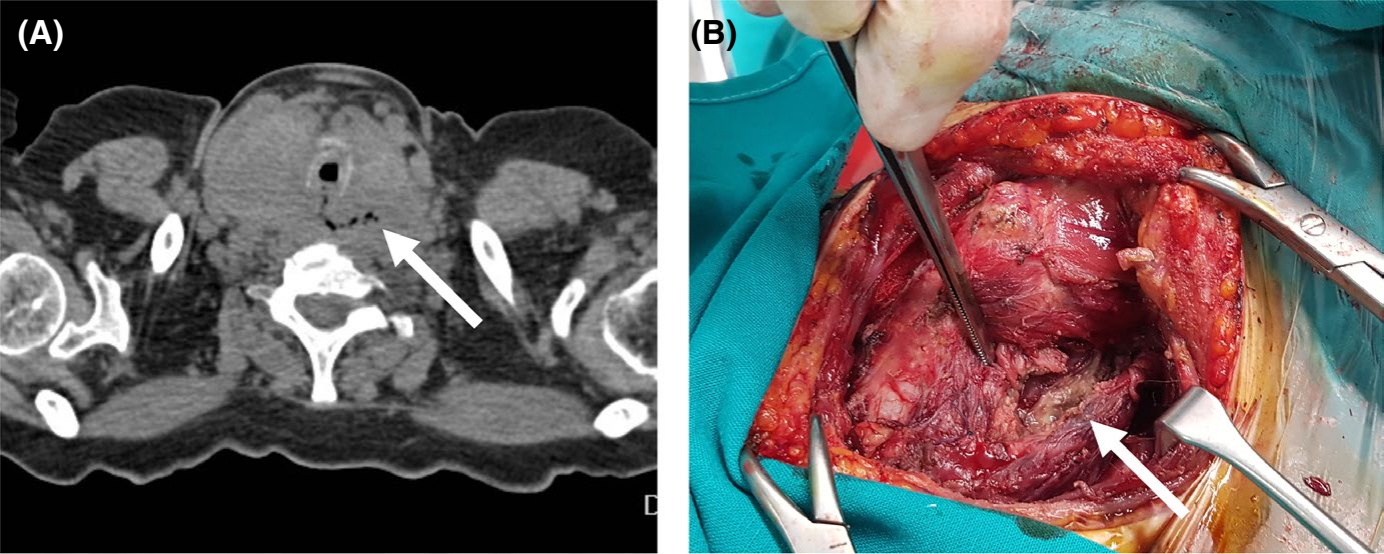
A, CT scan showing substernal goiter, trachea compression, and air bubbles surrounding the esophagus (white arrow). B, Intraoperative image showing perforation of the esophagus due to the thyroid lymphoma (white arrow)

A 65‐year‐old Caucasian woman was admitted due to anterior neck enlargement and compressive symptomatology for 2 months. Medical history only included rheumatoid arthritis. On physical examination, she had a stiff, nontender, and diffuse goiter. Laboratory investigation showed: thyroid‐stimulating hormone: 3.1 mIU/L (normal range 0.4‐4), 25‐hydroxy‐vitamin D: 12 ng/mL (normal range: 30‐60), parathyroid hormone: 85 pg/mL (normal range 15‐60), calcitonin: 2 pg/mL (normal range: <11.5), and thyroid auto‐antibodies: negative. Ultrasound showed a heterogeneous substernal goiter and a 34 mm hypoechoic nodule of irregular margins, in right thyroid lobe. Fine‐needle aspiration biopsy was inconclusive (Bethesda III). CT scan showed severe tracheal compression, esophageal dilatation, and air bubbles in the surrounding structures (Figure 1A, arrow). Differential diagnosis included follicular carcinoma, medullary carcinoma, anaplastic carcinoma, and thyroid lymphoma.

## 2 DISCUSSION AND OUTCOMES

The patient's swallowing capacity was progressively deteriorating. She underwent total thyroidectomy, revealing esophageal perforation (Figure 1B, arrow). Histological diagnosis revealed high malignancy diffuse large B‐cell lymphoma (DLBCL). The patient remained euthyroid after surgery. Chemotherapy was not feasible due to the patient's clinical state (acute renal failure and repeated respiratory infections). She died one month later.

Primary thyroid lymphoma (PTL) represents about 1%‐5% of thyroid malignancies.^1^ The most common subtype is DLBCL.^1^ Εsophageal perforation is a rare complication of PTL, characterized by poor prognosis.^2^ To the best of our knowledge, this is the second case reported in the literature.

## CONFLICT OF INTEREST

The authors declare that they have no conflict of interest regarding the publication of this case report.

## AUTHOR CONTRIBUTIONS

PA: wrote the paper, conceived and designed the case report. KV: analyzed and interpreted the data. KT: contributed to the analysis. EK: provided scientific input and approved the final version of the paper.
